# Inhibition of A5 Neurons Facilitates the Occurrence of REM Sleep-Like Episodes in Urethane-Anesthetized Rats: A New Role for Noradrenergic A5 Neurons?

**DOI:** 10.3389/fneur.2012.00119

**Published:** 2012-07-26

**Authors:** Victor B. Fenik, Vitaliy Marchenko, Richard O. Davies, Leszek Kubin

**Affiliations:** ^1^Department of Animal Biology, School of Veterinary Medicine, University of PennsylvaniaPhiladelphia, PA, USA

**Keywords:** clonidine, carbachol, hippocampus, hypoglossal motoneurons, pons, REM sleep, respiratory rhythm, theta rhythm

## Abstract

When rapid eye movement (REM) sleep occurs, noradrenergic cells become silent, with the abolition of activity in locus coeruleus (LC) neurons seen as a key event permissive for the occurrence of REM sleep. However, it is not known whether silencing of other than LC noradrenergic neurons contributes to the generation of REM sleep. In urethane-anesthetized rats, stereotyped REM sleep-like episodes can be repeatedly elicited by injections of the cholinergic agonist, carbachol, into a discrete region of the dorsomedial pons. We used this preparation to test whether inhibition of ventrolateral pontine noradrenergic A5 neurons only, or together with LC neurons, also can elicit REM sleep-like effects. To silence noradrenergic cells, we sequentially injected the α_2_-adrenergic agonist clonidine (20–40 nl, 0.75 mM) into both A5 regions and then the LC. In two rats, successful bilateral clonidine injections into the A5 region elicited the characteristic REM sleep-like episodes (hippocampal theta rhythm, suppression of hypoglossal nerve activity, reduced respiratory rate). In five rats, bilateral clonidine injections into the A5 region and then into one LC triggered REM sleep-like episodes, and in two rats injections into both A5 and then both LC were needed to elicit the effect. In contrast, in three rats, uni- or bilateral clonidine injections only into the LC had no effect, and clonidine injections placed in another six rats outside of the A5 and/or LC regions were without effect. The REM sleep-like episodes elicited by clonidine had similar magnitude of suppression of hypoglossal nerve activity (by 75%), similar pattern of hippocampal changes, and similar durations (2.5–5.3 min) to the episodes triggered in the same preparation by carbachol injections into the dorsomedial pontine reticular formation. Thus, silencing of A5 cells may importantly enable the occurrence of REM sleep-like episodes, at least under anesthesia. This is a new role for noradrenergic A5 neurons.

## Introduction

The rapid eye movement (REM) stage of sleep is a state characterized by wake-like cortical and hippocampal activation that occurs in the absence of awareness of the external environment, with tonic suppression of motor activity, random twitches of distal muscles, REMs, and distinct variability of breathing and blood pressure (Aserinsky and Kleitman, 1953). The state is initiated and orchestrated by a network of neurons located within the brainstem, as indicated by the classic lesion and trans-section experiments (Jouvet, 1962, 1994), but its occurrence is modulated by descending inputs, especially those from the posterior hypothalamus (Lin et al., 1989; Chemelli et al., 1999; Lu et al., 2007). Single cell recordings from noradrenergic neurons of the locus coeruleus (LC) and serotonergic neurons of the dorsal raphe nucleus revealed that REM sleep is associated with the silencing of these cells (McGinty and Harper, 1976; Trulson and Jacobs, 1979; Aston-Jones and Bloom, 1981) and concurrent activation of a subset of dorsal pontine cholinergic neurons (El Mansari et al., 1989; Steriade et al., 1990; Kayama et al., 1992; Thakkar et al., 1998). Based on these data and the studies showing that muscarinic cholinergic agonists delivered to the dorsomedial pontine tegmentum trigger a REM sleep-like state (Silberman et al., 1980; Baghdoyan et al., 1984; Kimura et al., 1990), a reciprocal model of the generation of REM sleep has been proposed that had as its key feature direct or indirect mutually inhibitory interactions between brainstem aminergic and cholinergic neurons (McCarley and Hobson, 1975). In the original model and its subsequent refinements, LC was seen as the key noradrenergic cell group whose silencing was necessary for the occurrence of REM sleep (McCarley, 2004). Consistent with the reciprocal interaction model, direct activation of LC neurons (Horvath et al., 1999; Bourgin et al., 2000; Carter et al., 2010) and adrenergic drug microinjections into the dorsal pontine tegmentum (Tononi et al., 1991; Cirelli et al., 1992; Bier and McCarley, 1994) impede the generation of REM sleep or abolish it altogether. However, lesions of the LC had only small and transient effects on the amount and pattern of REM sleep (Roussel et al., 1976; Webster and Jones, 1988; Blanco-Centurion et al., 2004), suggesting that the absence of LC activity is not a key prerequisite for the occurrence of REM sleep.

Recordings from noradrenergic cells of the pontine subcoeruleus region (Reiner, 1986) and the ventrolateral pontine A5 group (Fenik et al., 2002) revealed that, like LC neurons, these cells are also silenced during REM sleep. In addition, indirect evidence based on c-Fos immunohistochemistry suggested that both pontine A7 neurons and medullary A2 neurons also have reduced or abolished activity during REM sleep (Rukhadze et al., 2008). Serotonergic neurons of the dorsal raphe nucleus and serotonergic medullary neurons of the raphe obscurus and pallidus nuclei are also silenced during REM sleep (Heym et al., 1982; Woch et al., 1996). Whether silencing of all these neurons is necessary for the occurrence of REM sleep has not been tested, nor were any attempts made to determine whether any one of these groups plays a particularly important permissive role in the generation of REM sleep.

Over the years, we have extensively explored and validated an acute model of REM sleep-like state based on urethane-anesthetized rats. In this model, pontine microinjections of a cholinergic agonist, carbachol, trigger a state that is electrophysiologically similar to REM sleep and comprises activation of cortical EEG, generation of hippocampal theta-like rhythm, suppression of motoneuronal activity, and silencing of noradrenergic cells of the LC and A5 group (Kubin, 2001; Fenik et al., 2002, 2005; Fenik and Kubin, 2009). These REM sleep-like episodes last 2–4 min and can be elicited from the same site multiple times within the course of an acute experiment (Fenik et al., 2004; Rukhadze et al., 2008). As with natural REM sleep (Adamantidis et al., 2007), activation of cells in the wakefulness-promoting region of the posterior hypothalamus abolishes the ability of pontine carbachol to elicit REM sleep-like episodes in urethane-anesthetized rats (Lu et al., 2007). Also as in chronically instrumented, behaving rats (Pollock and Mistlberger, 2003; Sanford et al., 2003), dorsomedial pontine injections of the GABA_A_ receptor antagonist bicuculline trigger REM sleep-like episodes in urethane-anesthetized rats (Fenik and Kubin, 2009). These similarities demonstrate that important parts of the neuronal network responsible for the generation of REM sleep can be activated in a stereotyped manner under urethane anesthesia.

In the present study, we tested whether inhibition of activity in selected groups of pontine noradrenergic neurons elicits REM sleep-like effects in urethane-anesthetized rats, thus activating the brainstem REM sleep-generating network in a manner similar to the way it is activated by dorsomedial pontine microinjections of carbachol. We used microinjections of the α_2_-adrenergic agonist, clonidine, to silence pontine noradrenergic neurons. Noradrenergic neurons of the A5 group are located within a well-defined region of the caudal, ventrolateral pons between the lateral superior olivary nucleus and the roots of the seventh cranial nerve. To date, they have been seen as important for cardiorespiratory control and modulation of nociception (Hilaire et al., 1989; Huangfu et al., 1991; Clark and Proudfit, 1993; Erickson and Millhorn, 1994; Koshiya and Guyenet, 1994; Martin et al., 1999). However, A5 neurons also have efferent connections with brainstem regions important for the control of sleep-wake states, including the ventrolateral periaqueductal gray (Byrum and Guyenet, 1987; Kwiat and Basbaum, 1990), nucleus prepositus hypoglossi (Iwasaki et al., 1999), dorsomedial pontine REM sleep-triggering region (Semba, 1993), and medullary reticular paragigantocellular nucleus (van Bockstaele et al., 1989). In an earlier study (Fenik et al., 2002), we observed that clonidine injections into the A5 region did not have any tonic effects on activity of hypoglossal (XII) motoneurons or hippocampal activity but triggered in some animals transient hippocampal and motoneuronal changes similar to the REM sleep-like episodes elicited by pontine carbachol. Therefore, our goal was to investigate whether inhibition of pontine A5 cells, with or without concurrent inhibition of LC neurons, facilitates the occurrence REM sleep-like episodes in urethane-anesthetized rats. A preliminary report has been published (Kubin et al., 2003).

## Materials and Methods

### Animal preparation

Experiments were performed on 16 adult, male Sprague-Dawley rats [body weight: 376 ± 7 g (standard error – SE)] obtained from Charles River Laboratories (Wilmington, MA, USA). All animal procedures were approved by the Institutional Animal Care and Use Committee of the University of Pennsylvania and followed the guidelines for the care and use of experimental animals established by the National Institutes of Health.

The animals were pre-anesthetized with isoflurane (2%) and then anesthetized with urethane (1 g/kg, i.p., supplemented by 40 mg i.v. injections as needed). The trachea was intubated and a femoral artery and vein were catheterized for arterial blood pressure monitoring and fluid injections, respectively. The right XII nerve was cut and its central end placed inside a cuff electrode for recording (Fenik et al., 2001). The cervical vagi were cut to enhance XII nerve activity and eliminate its reflex modulation by pulmonary afferents. The head of the animal was placed in a stereotaxic holder, openings were made bilaterally in the interparietal bone and the dura removed for inserting a drug-containing pipette into the pons. Another opening was made for inserting a bipolar recording electrode into the hippocampus. The electrode was made from two Teflon insulated platinum wires (0.002″/0.004″ bare/coated diameter, A-M Systems, Carlsborg, WA, USA) with tips separated by 0.8 mm and was inserted 3.7 mm posterior to bregma, 2.2 mm to the right from midline and 2.4 mm below the cortical surface. Two screws were attached to the skull (2 mm anterior and 2 mm to the right, and 3 mm posterior and 2 mm to the left, of bregma) to record the electroencephalogram (EEG).

The animals were paralyzed with pancuronium bromide (2 mg/kg i.v., supplemented with 1 mg/kg injections as needed) and artificially ventilated with a mixture of air and oxygen (30–60% O_2_) at ∼50–60 lung inflations/min. After paralysis, the level of anesthesia was assessed by intermittently applying a pinch to the hind limb while recording the arterial blood pressure, EEG, hippocampal, and XII nerve activity. Absence of pinch-induced changes in either the respiratory rate or XII nerve activity and only transient changes in blood pressure, EEG, and hippocampal signals, similar to those before paralysis, indicated an adequate level of anesthesia. Rectal temperature was maintained at 36–37°C throughout the experiments. The end-expiratory CO_2_ (Columbus Instruments capnograph, Columbus, OH, USA) was adjusted at the beginning of the experiment to maintain a steady respiratory modulation of XII nerve activity and then kept constant.

### Electrophysiological recordings

Electroencephalogram (bandwidth 0.5–100 Hz), hippocampal activity (2–20 Hz), XII nerve activity, and extracellularly recorded single cell activity (30–2500 Hz) were amplified with AC amplifiers (N101, Neurolog System; Digitimer, Hertfordshire, UK). The signals were continuously monitored on an 8-channel chart recorder (TA-11; Gould Instruments, Valley View, OH, USA) and stored using a 16-channel digital tape recorder (C-DAT; Cygnus Technology, Delaware Water Gap, PA, USA) together with tracheal pressure, end-expiratory CO_2_, arterial blood pressure, and an event marker. XII nerve activity was fed into a moving average circuit (MA-821 RSP; CWE, Inc., Ardmore, PA, USA; time constant: 100 ms).

### Drug microinjections

A glass pipette (A-M Systems) with a tip diameter of 25–30 μm was filled with the α_2_-adrenergic agonist clonidine (Sigma, St. Louis, MO, USA; 0.75 mM in 0.9% NaCl prepared from frozen aliquots before each experiment). In separate experiments, pipettes were filled with carbamylcholine chloride (carbachol; Sigma; 10 mM in 0.9% NaCl). Pontamine sky blue dye (2%, ICN Biomedicals, Inc., Aurora, OH, USA) was added to these solutions to mark the injection sites. Clonidine injections into the A5 region had a volume of 20–40 nl each, and those placed in the LC 20 nl. The target stereotaxic coordinates for the A5 group were 9.8 mm posterior, ±2.7 mm lateral and 9.9 mm ventral relative to bregma, according to a rat brain atlas (Paxinos and Watson, 1997). For the LC, they were 9.7 mm posterior, ±1.2 mm lateral, and 6.8 mm ventral. Carbachol injection volumes were 10 nl; the locations of the effective injection sites were published previously (Fenik et al., 2005; Fenik and Kubin, 2009). The drugs were injected over 10–30 s by applying pressure to the fluid in the pipette while movement of the meniscus was monitored with 2 nl resolution through a calibrated microscope.

### Experimental protocol

Once the animal was prepared for recording, at least three penetrations were made with a tungsten recording microelectrode (4–5 μm tip exposure) to map out the location of one LC. LC cells were recognized based on their firing rates at 1–5 Hz, a characteristic configuration of their action potentials, brisk activation in response to a noxious pinch of the hindlimb followed by a silent period (Fenik et al., 2002), and location adjacent to large cells of the mesencephalic trigeminal nucleus, which are strongly activated by tactile stimuli applied to the muzzle region. Once the location of one LC was established, the nominal coordinates for A5 and LC described above were re-calculated relative to this site. This approach allowed us to achieve proper placement of microinjections in A5 and, if needed, also LC in most animals, as subsequently histologically verified.

After the mapping of LC location and allowing a 10-min period to verify that all signals were stable, a clonidine-containing pipette was inserted into the A5 region on one side and 20–40 nl of clonidine solution were injected after an additional 60–90 s. The pipette was then moved to the A5 region on the opposite side and another injection made. In some experiments, if no changes occurred within the next 4 min, the pipette was inserted into one LC and a 20-nl injection made. If this LC injection was also without effect, an additional one was placed in the LC of the opposite side. Successive injections were separated on the average by 420 ± 25 s (range: 300–640 s), which allowed sufficient time to determine whether any one of them initiated REM sleep-like, or any other, changes in the recorded signals. If changes were noted within this time frame, no further injections were made and observation continued until a recovery occurred from the effect of the last injection. In three rats, injections were made only into the LC, without any subsequent injections into the A5 regions. The reason for not continuing with A5 injections in these experiments was the evidence that α_2_-adrenergic agonists injected into the dorsomedial pontine tegmentum, which is located adjacent to the LC, inhibit the generation of REM sleep (Tononi et al., 1991) and the evidence that cells located ventral to LC express α_2_-adrenergic receptors (Volgin et al., 2009). A spread of clonidine from the LC to the adjacent dorsomedial pontine reticular formation would likely occur over the 10–15 min needed to place additional injections in the A5 regions, resulting in inhibition of dorsomedial pontine reticular neurons essential for the generation of REM sleep. Therefore, injections into the A5 region were not made when the LC was targeted first because any negative results with subsequent clonidine injections into the A5 regions would be inconclusive.

The amplitude of XII nerve activity and central respiratory rate were measured from the moving average of nerve activity over 30–60 s control period prior to each injection and over another 30 s period centered on the peak of the response, if it occurred. The onset and offset times of the effects were identified as the times when a 10% decrease from baseline XII nerve activity and a 50% recovery occurred, respectively. For spectral analysis of the EEG and hippocampal activity, the signals were digitized with 100 Hz sampling rate using Spike-2 software (Cambridge Electronics Design, UK) and the power spectrum was then calculated within 0–20 Hz range over a 60- to 90-s control period and a 60- to 90-s period centered on the peak of the effect.

### Histological verification of the injection sites

At the conclusion of the experiment, the animal received an additional dose of urethane (1 g/kg, i.v.) and heparin (100 units) and was intra-arterially perfused with cold 0.9% NaCl followed by 10% phosphate-buffered formalin. The brainstem was removed, post-fixed in the same fixative, transferred to 30% sucrose and then cut on a cryostat into 100 μm coronal sections. All sections containing Pontamine sky blue dye were serially mounted and stained with Neutral red to localize the injection sites. The centers of the injection sites were then transferred onto the nearest standard cross-sections from a rat brain atlas (Paxinos and Watson, 1997).

### Statistical analysis

After verification that the data were normally distributed, a two-tailed paired Student’s *t*-test was used for comparison of measurements taken at different times before and after clonidine injections (SigmaStat, Jandel, Inc., San Rafael, CA, USA). The variability of the means is characterized by the SE throughout the report. Differences were considered significant when *p* < 0.05.

## Results

### REM sleep-like episodes elicited by clonidine injected into the A5 regions, or A5 regions followed by LC

**Figure 1:**
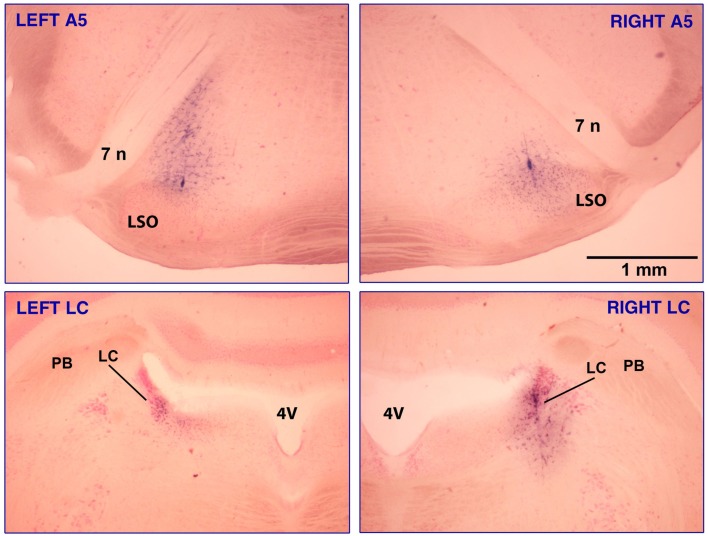
**Clonidine injection sites in the A5 and LC regions in one of the rats that had injections made into all four sites, A5 bilaterally (top panels) and LC bilaterally (bottom panels)**. The sites are marked with Pontamine sky blue dye added to the clonidine solution. The injection volumes were 30 nl per side for the A5 region and 20 nl per side for LC. In the A5 region, most noradrenergic cells are located between the seventh nerve (7 n) and the lateral superior olivary nucleus (LSO) and some also occur along the dorsolateral border of the LSO (Fenik et al., 2002). Intense blue staining marking the track of the injection pipette can be seen in the bottom right and both top panels. The calibration bar in the top-right panel applies to all panels. Abbreviations: 4V, 4th ventricle; PB, parabrachial region.

Figure 1 shows brain sections from one rat in which injections were placed in all four sites, A5 bilaterally, and then LC bilaterally, with the sites marked by deposits of Pontamine sky blue included in the clonidine solution.

**Figure 2:**
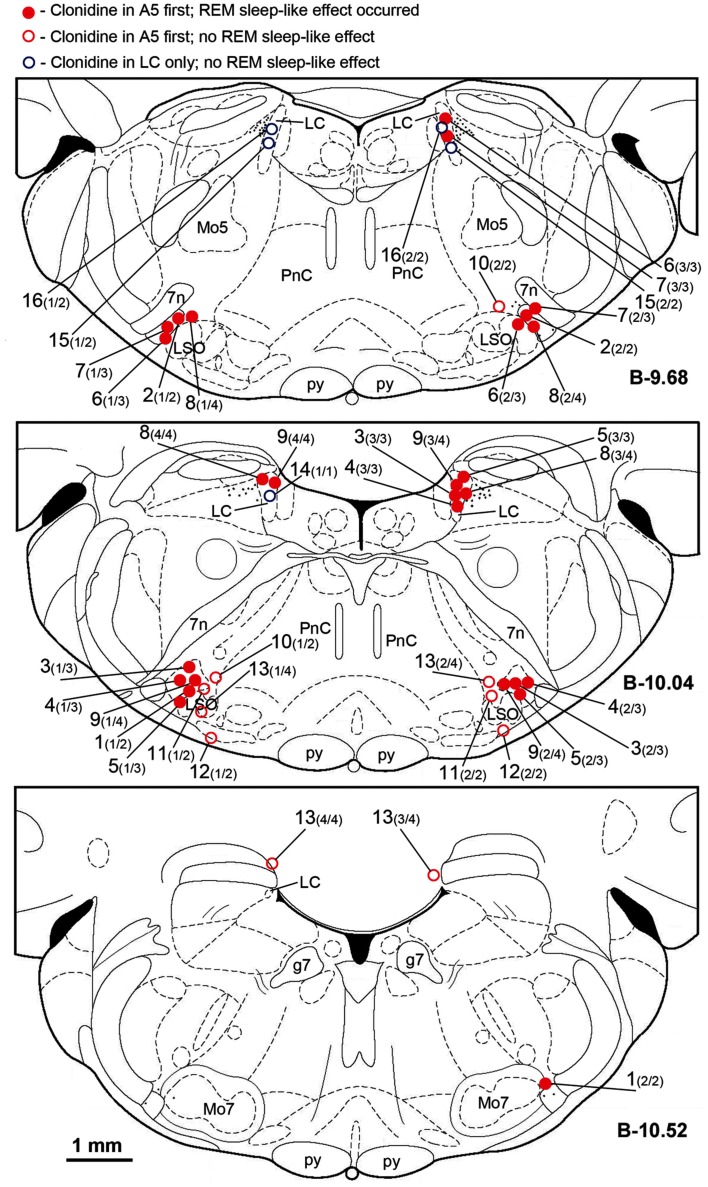
**Location of the sites of all clonidine injections made in this study**. Each symbol represents the center of a separate injection site, as verified histologically from the location of Pontamine blue dye deposit (see Figure 1). Injection sites were superimposed onto the closest standard transverse brain section from a rat brain atlas (Paxinos and Watson, 1997) at the indicated levels measured from bregma (B). Each injection site is marked by the experiment (animal) number followed in parenthesis by the sequential order of the injection and the total number of sites injected in this experiment. Red filled and open circles correspond to the experiments in which clonidine was injected first into the A5 regions bilaterally and then, in some animals, also into the locus coeruleus (LC) uni- or bilaterally. Open blue circles correspond to the three experiments in which clonidine was injected into the LC only. Red filled circles indicate the experiments in which REM sleep-like episode occurred following clonidine injections; open symbols correspond to the experiments in which REM sleep-like episode did not occur at the end of the injection sequence (all three experiments with clonidine injections into the LC only and four experiments in which clonidine was first injected into the A5 region bilaterally but the injections were placed medial to the site where most noradrenergic A5 neurons are located). The injection sites illustrated in Figure 1 correspond to experiment 9 in this figure. Abbreviations: 7n, 7th cranial nerve; g7, genu of the facial nerve; Mo5, trigeminal motor nucleus; LC, locus coeruleus; LSO, lateral superior olivary nucleus; Mo7, facial motor nucleus; p, pyramidal tract; PnC, nucleus pontis caudalis.

Figure 2 shows the locations of all clonidine injection sites in all 16 animals. Each injection site is labeled with the animal number followed in parenthesis by the sequential number of the injection and the total number of injections made in this animal (up to 4, if both A5 regions and both LC were targeted). In two rats, clonidine was successfully injected into the A5 regions bilaterally (experiments 1 and 2; filled red circles). In seven rats, successful injections were successively made into the A5 regions bilaterally and then into one or both LC (experiments 3 through 9). In three rats, the A5 regions were targeted bilaterally but the centers of the injections were then found medial to the A5 region (experiments 10–12; open red circles), and in three rats, clonidine was injected into the LC only (experiments 14–16; open blue circles). In the following description of our results, we refer to the corresponding experiment numbers, as shown in Figure 2.

**Figure 3:**
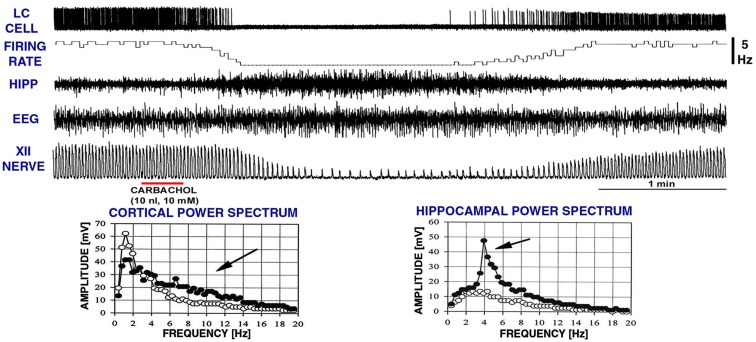
**A typical REM sleep-like episode elicited by carbachol injection into the dorsomedial pontine tegmentum while recording extracellularly from a noradrenergic cell of the LC**. Characteristic of these episodes are reduction of low-frequency components in favor of higher frequencies (6–12 Hz) in the power spectrum of cortical electroencephalogram (EEG), the appearance of theta-like rhythm in the hippocampus (HIPP; 3–4 Hz under anesthesia; Vertes et al., 1993; c.f., graphs at the bottom), reduction of inspiratory activity of the hypoglossal (XII) nerve, and slowing of the respiratory rate. The top two traces show that, when these changes occur, noradrenergic LC cells stop firing. The episodes typically last 2–4 min. The record was obtained in a related study (Fenik et al., 1999). XII nerve trace shows integrated activity of the XII nerve, with each upward deflection corresponding to the inspiratory phase of the central respiratory cycle. The power spectra were calculated for a 60-s period of activity prior to carbachol injection (open circles) and for a 60-s interval centered on the peak of the response to carbachol (filled circles). Arrows point to the parts of the spectra where differences can be noted between the control period and the period of REM sleep-like episode.

Our goal was to investigate the ability of clonidine injections into the A5 and LC to elicit REM sleep-like episodes as those elicited by carbachol in urethane-anesthetized rats that we have previously characterized as electrophysiologically analogous to naturally occurring REM sleep (Fenik et al., 2002, 2005; Lu et al., 2007; Fenik and Kubin, 2009; see Kubin, 2001; Kubin and Fenik, 2004 for reviews). Therefore, Figure 3 shows, for reference, a typical carbachol-elicited REM sleep-like episode during which we also recoded from a LC neuron. Characteristic of the carbachol-elicited REM sleep-like episodes are the gradual depression and then recovery of inspiratory activity of the XII nerve accompanied by slowing of the respiratory rate, appearance of a theta-like rhythm in the hippocampus (3–4 Hz, when elicited under anesthesia; Vertes et al., 1993), a rightward shift of the peak in EEG power spectrum, and often an elevation of EEG power in the 6–12 Hz range). As shown in Figure 3, LC cells are silenced (Fenik et al., 1999; Kubin, 2001), as are noradrenergic neurons of the A5 group (Fenik et al., 2002). The carbachol-induced episodes maintain the same characteristics when elicited repeatedly within the course of an acute experiment, with only their duration typically varying from 2 to 4 min, and at most from 1.5 to 10 min (Rukhadze et al., 2008).

**Figure 4:**
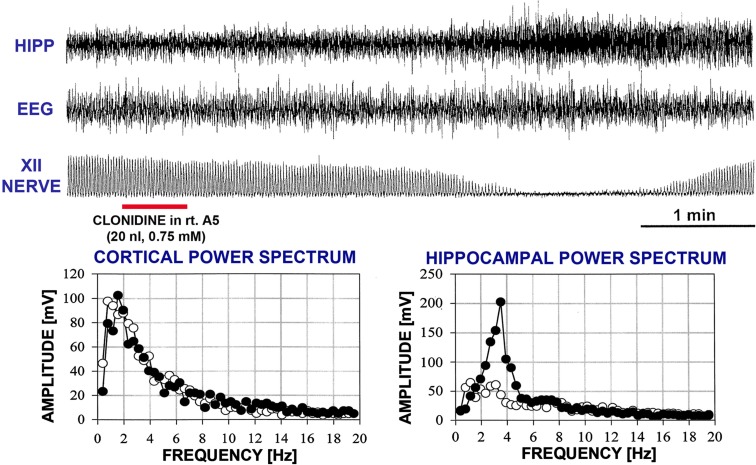
**A REM sleep-like episode triggered after clonidine was injected into the A5 region on the left and then right side**. The injection marker shows the second of the two injections. Trace labels and figure format are the same as for Figure 3. In this case, inspiratory activity of the XII nerve was transiently abolished at the peak of the response. One can also note that the central respiratory rate was reduced just before the disappearance of XII nerve activity and just after it reappeared when compared with the pre-injection period. The power spectra for the control period and the period centered on the peak of the response are shown with open and filled circles, respectively.

In two rats, REM sleep-like episodes with the same characteristics as those produced by dorsomedial pontine injections of carbachol occurred after bilateral clonidine injections into the A5 regions, and no additional injections were made in these animals (experiments 1 and 2 in Figure 2). The episodes started 160–170 s after the onset of the second clonidine injection and lasted 150–230 s. Figure 4 shows hippocampal, EEG, and XII nerve activities recorded before and after the second of the two clonidine injection and the power spectra of the cortical and hippocampal signals calculated over 90 s intervals positioned before and at the peak of the clonidine effect. In this rat (experiment 1 in Figure 2), XII nerve activity was transiently abolished at the peak of the response, as is also sometimes the case with the episodes elicited by pontine carbachol (Fenik et al., 2002, 2005; Fenik and Kubin, 2009). A gradual slowing of the respiratory rate, from 40 to 30 min^−1^, is evident prior to the disappearance of XII nerve activity.

**Figure 5:**
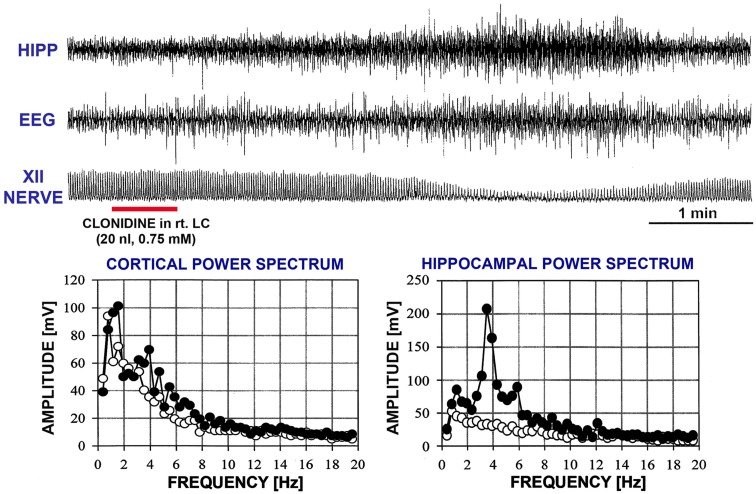
**A REM sleep-like episode triggered after clonidine was injected into the A5 regions bilaterally and then into the right LC**. The injection marker shows the LC injection only. Trace labels and figure format are the same as for Figure 3. Note the similarity of the changes elicited here by clonidine to those elicited by carbachol and illustrated in Figure 3. The power spectra for the control period and the period centered on the peak of the response are shown with open and filled circles, respectively.

In another five rats, within 7 min after bilateral clonidine injections into the A5 regions that did not cause any changes, a third clonidine injection was made into one LC (experiments 3 through 7 in Figure 2). In these rats, the third (LC) injection elicited a REM sleep-like episode. The effect started 72–240 s (150 ± 33 s) after the injection into the LC and lasted 155–255 s (204 ± 19 s). The inspiratory activity of the XII nerve was reduced to 27–46% of the control level (34.6 ± 3.3%; *p* = 0.0004) and the respiratory rate decreased by 5–28 min^−1^ (13.0 ± 4.1 min^−1^, or from 46.8 ± 1.8 to 33.8 ± 5.0 min^−1^; *p* = 0.04). Figure 5 shows hippocampal, EEG, and XII nerve activities recorded in one of these five rats before and after clonidine injection into one LC that was preceded by bilateral clonidine injections into the A5 regions that alone had no effect (experiment 3 in Figure 2).

In an additional two rats, bilateral clonidine injections into both A5 and LC were necessary to elicit the REM sleep-like episode, i.e., the episode occurred only after a clonidine injection into the LC that was preceded by bilateral clonidine injections into the A5 regions and a clonidine injection into the LC on the opposite side (experiments 8 and 9 in Figure 2). These episodes started 73–82 s after the last injection and lasted 173–319 s. During one of them, XII nerve activity was transiently abolished and during the other one it was reduced to 55% of the control level, and respiratory rate was reduced from 41 min^−1^ to less than 25 min^−1^ and from 44 to 34 min^−1^, respectively.

**Figure 6:**
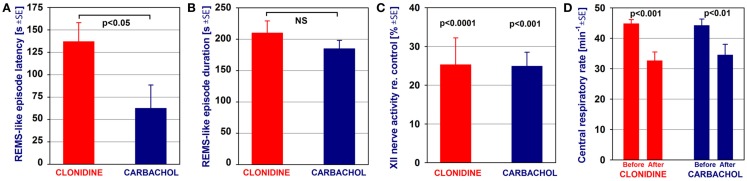
**Comparison of the average characteristics of the REM sleep-like episodes elicited by clonidine injections into the A5 regions, with or without additional injections into the LC, and those elicited by carbachol injections into the dorsomedial pontine tegmentum**. The episodes elicited by clonidine occurred with a significantly longer latency than those elicited by carbachol **(A)**. Other than that, neither the episode duration **(B)**, nor the magnitude of suppression of XII nerve activity **(C)**, nor the reduction of the central respiratory rate **(D)** were significantly different between the episodes elicited by clonidine and carbachol. The bars show a comparison of the average data from nine REM sleep (REMS)-like episodes elicited by clonidine in nine rats in this study and average data from six REMS-like episodes elicited by pontine carbachol (10 nl, 10 mM) in six rats of one of our previous studies (Fenik et al., 2005). The significance levels shown in **(C,D)** refer to the differences between the measurements taken prior to and during the REMS-like episode. NS, not statistically significant.

Figure 6 compares the average characteristics of all nine REM sleep-like episodes elicited by clonidine injections into both A5 regions, with or without additional injections into the LC, obtained from nine rats in this study with the average effects of carbachol injections into the dorsomedial pons made in six urethane-anesthetized, paralyzed and artificially ventilated rats in one of our earlier studies (Fenik et al., 2005). The episodes elicited by clonidine occurred with a significantly longer latency than those triggered by carbachol, 137 ± 21 vs. 63 ± 26 s (Figure 6A). Other than that, neither the episode duration (210 ± 19 vs. 185 ± 13 s; Figure 6B), nor the magnitude of suppression of XII nerve activity (to 25.3 ± 6.9% of the control level vs. 24.9 ± 3.6%; Figure 6C), nor the reduction of the central respiratory rate (44.8 ± 1.3 to 32.6 ± 2.9 vs. 44.2 ± 2.1 to 34.5 ± 3.5 min^−1^; Figure 6D) were significantly different. It is of note that, similar to the effects of dorsomedial pontine carbachol injections, the REM sleep-like effects elicited by clonidine injected into the A5 and LC regions were of an all-or-none type; i.e., clonidine injections had no effect on the EEG, hippocampal or XII nerve activity or respiratory rate unless they elicited a stereotyped REM sleep-like episode.

### Clonidine injections into LC only or sites adjacent to the A5 region did not elicit REM sleep-like episodes

In three rats, clonidine injections were placed into LC only, bilaterally in two, and unilaterally in one, animal (experiments 14, 15, and 16 in Figure 2). None of these animals produced REM sleep-like episodes. In another three rats, REM sleep-like episodes did not occur when clonidine was injected aiming at both A5 regions but subsequent histological verification indicated that the injections were placed medial to the A5 regions (experiments 10, 11, and 12 in Figure 2). No additional injections were made in these rats. In another rat, we intended to place clonidine injections into the A5 region bilaterally and then into LC bilaterally but, for technical reasons, we missed all targets by 500–900 μm (experiment 13 in Figure 2). Also in this rat no REM sleep-like effects occurred. Thus, in two animals of the present study, bilateral injections that properly hit the A5 region were sufficient to elicit REM sleep-like episodes, in most experiments, bilateral clonidine injections into the A5 region and into at least one LC were needed, whereas uni- or bilateral injections into the LC only or into other than LC or A5 pontine sites were ineffective.

## Discussion

Although all studied to date noradrenergic neuronal groups of the pons cease firing during REM sleep, it has not been determined whether all of them need to be silenced for the REM sleep to occur. Alternatively, the silencing of some groups may be necessary whereas the silencing of other groups may be not. Traditionally, the LC has been seen as the key noradrenergic group that has to cease firing for REM sleep to occur because it is the most numerous group and because activation of LC neurons effectively suppresses generation of REM sleep. The results of our study do not contradict the findings that activation of LC neurons can effectively suppress REM sleep, but they point to an important permissive role of the silencing of noradrenergic A5 neurons. To date, A5 neurons have been proposed to contribute to cardiorespiratory regulation and nociception, whereas their role as an important part of the brainstem network responsible for the generation of REM sleep has not been considered. Based on our results, we propose that silencing of noradrenergic A5 neurons is necessary for the occurrence of REM sleep.

Our results support an important permissive role of A5 neurons in the generation of REM sleep because of the following. In two experiments, we observed that bilateral clonidine injections into the A5 regions were sufficient to initiate REM sleep-like episodes in our rat model, whereas REM sleep-like episodes did not occur in any of the three rats in which clonidine was injected into the LC only and in another three rats in which only A5 regions were targeted but the injections were placed too medial. Furthermore, in most other rats, REM sleep-like episodes were triggered when clonidine was properly placed bilaterally into the A5 regions and into only one LC. Since in these experiments LC activity on the opposite side was not pharmacologically inhibited, one must conclude that the persistence of LC activity on the intact side was not sufficient to prevent generation of the REM sleep-like episodes when A5 neurons were bilaterally inhibited. This is rather remarkable if one considers that the LC contains nearly five times more noradrenergic neurons that the A5 group (Rukhadze and Kubin, 2007) and that the compact size of the LC makes it relatively easier to deliver clonidine by microinjection to all its neurons than to achieve the same for the A5 group due to its considerably larger antero-posterior and medio-lateral dimensions.

The urethane-anesthetized rat offers an attractive model with which to study the effects of various manipulations that remove selected inputs that are normally associated with wakefulness or arousal and oppose the occurrence of REM sleep. In urethane-anesthetized rats, dorsomedial pontine injections of carbachol readily induce REM sleep-like episodes (Kubin, 2001; Fenik et al., 2004, 2005; Lu et al., 2007; Fenik and Kubin, 2009), whereas, in unanesthetized, behaving rats, carbachol is less effective (Gnadt and Pegram, 1986; Okabe et al., 1998; Boissard et al., 2002). The difference may be due to concomitant REM sleep-suppressing cholinergic effects exerted in adjacent reticular regions and/or suprapontine influences that act to oppose cholinergic triggering of REM sleep in rats (Taguchi et al., 1992; Bourgin et al., 1995; Okabe et al., 1998; discussed by Kubin, 2001). Indeed, anesthetics may activate some of the same neuronal groups that are also activated during natural non-REM sleep (Lu et al., 2008) and deactivate certain cholinergic and GABAergic neurons in a manner analogous to that observed during non-REM sleep (Vanini et al., 2011). This may then favor activation of the brainstem network responsible for the generation of REM sleep. Occasionally, some urethane-anesthetized rats in our studies spontaneously generate REM sleep-like episodes like those elicited by dorsomedial pontine carbachol (or bicuculline) injections and like those observed in the present study following clonidine injections. Such spontaneous episodes, not related to any obvious external triggering event, are relatively rare but may occur more frequently following multiple pontine carbachol injections (Rukhadze et al., 2008). Thus, the urethane-anesthetized rats have an inherently high propensity to enter a REM sleep-like state. At the same time, this propensity probably varies among the preparations due to a host of subtle factors (e.g., level of anesthesia or brain perfusion). This variability may be the reason why, in some animals of the present study, silencing of A5 neurons only was sufficient to elicit REM sleep-like episodes, whereas in other rats inhibition of LC neurons on at least one side was needed to achieve this effect. Our present data show that activity of pontine noradrenergic neurons, including those of the A5 group, acts against spontaneous occurrence of REM sleep-like episodes in urethane-anesthetized rats.

The hypothesis that the silencing of LC neurons is a key factor enabling the occurrence of REM sleep has been previously questioned on the basis of limited decrements of c-Fos expression in LC neurons following periods of enhanced REM sleep produced as a result of prior REM sleep deprivation (Leger et al., 2009). Based on those findings, the authors suggested that the silencing of other noradrenergic neurons may be more important. Our results suggest that the A5 group is particularly important, which is somewhat unexpected because cardiorespiratory control and modulation of nociception are the main functions ascribed to A5 neurons (Sagen and Proudfit, 1986; Hilaire et al., 1989; Huangfu et al., 1991; Clark and Proudfit, 1993; Erickson and Millhorn, 1994; Koshiya and Guyenet, 1994; Martin et al., 1999; Bajic and Commons, 2010). Supportive of these functions are the descending projections of A5 neurons to the intermediolateral cell column of the thoracic spinal cord and the ventrolateral medullary A1 region (Loewy et al., 1979, 1986; Westlund et al., 1983; Byrum and Guyenet, 1987; Clark and Proudfit, 1993; Howorth et al., 2009; Marques-Lopes et al., 2010). It is, however, of note that other projections of A5 neurons target brainstem regions that are strongly associated with the control of sleep-wake states, such as the dorsal raphe nucleus (Peyron et al., 1996), ventrolateral periaqueductal gray (Byrum and Guyenet, 1987; Kwiat and Basbaum, 1990), nucleus prepositus hypoglossi (Iwasaki et al., 1999), dorsomedial pontine REM sleep-triggering region (Semba, 1993), and medullary reticular paragigantocellular nucleus (van Bockstaele et al., 1989). Furthermore, some A5 neurons have ascending projections to the paraventricular nucleus of the hypothalamus (Byrum and Guyenet, 1987) and paucisynaptic connections to the CA1 region of the hippocampus (Castle et al., 2005). Many of these connections are reciprocal, and many parallel similar connections of the LC. Through these connections A5 neurons may play an important role in the control of behavioral states.

Based on our results, we cannot exclude that clonidine injected into the A5 region facilitated the occurrence of REM sleep-like episodes through its actions on non-noradrenergic neurons located within, or adjacent to, the A5 region. However, there is currently no evidence for the presence in the A5 region of non-noradrenergic neurons that would have afferent and efferent connections appropriate for interaction with other brainstem regions important for the generation of REM sleep. Only the noradrenergic neurons located in the A5 region consistently express the inhibitory α_2A_-adrenergic receptors and are inhibited by low doses of clonidine (Byrum et al., 1984; Guyenet et al., 1994; Koshiya and Guyenet, 1994; Fenik et al., 2002). Furthermore, the REM sleep-like episodes were facilitated in our study only when clonidine injections were properly placed within the A5 region. In three rats in which clonidine was injected bilaterally medial to the region containing most noradrenergic A5 neurons (experiments 10–12 in Figure 2), REM sleep-like episodes did not occur. Also, clonidine injections like those conducted in the present study targeting the noradrenergic A7 group located in the lateral pons did not elicit REM sleep-like episodes (Fenik et al., 2008). Thus, the REM sleep-like effects observed in our present study were dependent on the presence of clonidine-receptive neurons and occurred when the injections were properly placed into the A5 region. This leaves little room for the possibility that inhibition of some not yet identified non-noradrenergic neurons located in the ventrolateral pons facilitated the occurrence of REM sleep-like episodes in our experiments.

The comparison of the different characteristics of the REM sleep-like episodes triggered by clonidine injected into the A5 and LC regions and carbachol injected into the dorsomedial pons (Figure 6) revealed that only the latency of the effect was significantly longer for clonidine than for carbachol. The reason for this may be our use of a rather low concentration of clonidine (0.75 vs. 10 mM for carbachol), a slightly higher molecular weight of clonidine than carbachol (230 vs. 183 g) which would cause slower diffusion, and also the volume of tissue occupied by noradrenergic A5 neurons which is probably larger than the carbachol-sensitive region in the dorsolateral pons (Fenik and Kubin, 2009). Other than the latency difference, the REM sleep-like episodes were nearly identical, suggesting that both clonidine and carbachol only triggered the REM sleep-like episodes, whereas their pattern and duration were controlled and orchestrated by a common brainstem REM sleep-generating neuronal network.

## Conclusion

Although the A5 group has not been implicated to date in the regulation of REM sleep, our results suggest that the silencing of noradrenergic A5 neurons is necessary for the generation of this state. Thus, the connections between A5 neurons and other brainstem neuronal groups involved in the generation of REM sleep need to be investigated in order to better understand the role of A5 neurons in the regulation of sleep-wake states.

## Conflict of Interest Statement

The authors declare that the research was conducted in the absence of any commercial or financial relationships that could be construed as a potential conflict of interest.
